# Therapeutic efficacy of rare earth carbonate with chemoradiotherapy in late-stage non-small cell lung cancer: a cohort prospective study

**DOI:** 10.3389/fendo.2023.1301032

**Published:** 2023-12-15

**Authors:** Qiang Cao, Xia Ye, Xinyan Wu, Qi Zhang, Junling Gong, Yuquan Chen, Yanwei You, Jun Shen, Yi Qiang, Guangzhu Cao

**Affiliations:** ^1^Department of Earth Sciences, Kunming University of Science and Technology, Kunming, China; ^2^Department of Pharmacy, Nanjing Drum Tower Hospital, Affiliated Hospital of Medical School, Nanjing University, Nanjing, China; ^3^Department of Oncology, The Affiliated Hospital of Youjiang Medical University for Nationalities, Baise, China; ^4^College of Veterinary Medicine, Sichuan Agricultural University, Chengdu, China; ^5^Undergraduate Department, Taishan University, Taian, China; ^6^School of Public Health, Nanchang University, Nanchang, China; ^7^Institute of Medical Information/Library, Chinese Academy of Medical Sciences, Beijing, China; ^8^Division of Sports Science & Physical Education, Tsinghua University, Beijing, China

**Keywords:** concurrent chemoradiotherapy, non-small cell lung cancer, immune escape, progression-free survival, lung cancer, prognostic factors

## Abstract

**Objective:**

To compare the therapeutic effects and adverse reactions of sterilizing rare earth carbonate combined with concurrent chemoradiotherapy and simple concurrent chemoradiotherapy in the treatment of late-stage non-small cell lung cancer (NSCLC), and to analyze the reasons for the differences.

**Method:**

A total of 817 patients with pathologically diagnosed late-stage NSCLC from June 1, 2021 to December 30, 2022, in the affiliated hospital of Kunming University of Science and Technology, were selected. They were randomly divided into a control group of 394 people and an experimental group of 423 people. The control group was given concurrent chemoradiotherapy (cisplatin + etoposide), while the experimental group simultaneously took a low dose of sterilized rare earth carbonate (0.05mg/Kg). The χ² test and Fisher’s test were used to compare the clinical pathological features, objective response rate (ORR), ECOG score, and adverse reactions of the two groups of patients, while survival analysis was used to compare the progression-free survival (PFS) of the two groups. Cox regression analysis was used to test factors related to prognosis.

**Results:**

The differences in clinical pathological features between the two groups of patients were not statistically significant, with all P>0.05. The ORR of the control group was 45.18% (178/394), and the experimental group was 89.83% (380/423), with a statistically significant difference (P=0.001). After treatment, the ECOG score of the experimental group was lower than that of the control group, P<0.001. The adverse reaction grading of patients in both groups was below level 3 after treatment, and no treatment-related fatalities occurred. The risk of pulmonary infection and bone marrow suppression in the experimental group was lower than that in the control group.

**Conclusion:**

In late-stage NSCLC patients, compared with simple concurrent chemoradiotherapy, the combination of concurrent chemoradiotherapy and sterilizing rare earth carbonate can significantly improve the short-term therapeutic effect and prognosis of patients, with good safety.

## 1 Introduction

Among the plethora of malignant neoplasms encountered in clinical oncology, pulmonary carcinoma (CA) prominently stands out. Data from 2020 elucidates that China bore witness to an estimated 715,000 fatalities attributed to this pernicious ailment, representing a staggering 40% of the global mortality rate from pulmonary carcinoma (1–3). The subtype known as Non-small cell lung cancer (NSCLC) predominates, constituting approximately 85% of the cases. In recent years, the incidence and mortality rates of NSCLC in various countries around the world have been on the rise, making NSCLC a significant disease that severely threatens global health (4–6). At the juncture of initial diagnosis, a significant proportion, approximately 30%, of individuals afflicted with NSCLC find themselves grappling with an advanced stage of the disease, tragically bypassing the window of opportunity for surgical intervention (7–9). The prevailing modality of treatment, subscribed to for stage III NSCLC patients bereft of surgical prospects, hinges on the synchronized application of chemotherapy and radiotherapy (10, 11). Despite the aggressive treatment regimen, the half-decade survival metrics offer a somber picture, delineating a survival rate that languishes between 25% and 30%. Sterilized rare earth carbonate (REC) is a mixture of rare earth carbonates and is widely used in various fields such as industry and medicine (12–15). Due to the high cost-effectiveness and strategic value of RCE, REC is considered an important resource in industrial production. Exploration of REC’s functionality has never ceased, and recent studies have found that REC has inhibitory effects on various cancers (16–18). Central to understanding the prognosis and guiding treatment strategies in non-small cell lung cancer (NSCLC) is the TNM staging system. TNM, an acronym for Tumor, Node, Metastasis, is a universally adopted classification system that describes the extent of cancer spread. The ‘T’ denotes the size and extent of the primary tumor, with higher numbers (T3, T4) indicating larger or more invasive tumors. This staging provides crucial information for the selection of appropriate therapeutic approaches and for understanding the potential challenges and prognostic implications in managing late-stage NSCLC.Several studies indicate that concurrent chemoradiotherapy combined with sterilized rare earth carbonate can significantly improve the short-term therapeutic effects and prognosis for cancer patients (19–21). Ding etal’s study (22) suggests that REC can be a potential drug for cancer. Dot etal’s study (23) has clinically proven that REC can inhibit PD-1 and PD-L1, thereby enhancing the activity of cytotoxic T lymphocytes (CTL), and subsequently preventing tumors from immune escape.

Currently, more and more scholars are combining low doses of REC in the treatment of various cancers to improve patient survival rates (24–26). Against this backdrop, the primary objective of this study is to evaluate the therapeutic efficacy and safety of sterilized rare earth carbonate combined with concurrent chemoradiotherapy in the treatment of late-stage NSCLC patients. Specifically, we hypothesize that the addition of REC to the standard treatment regimen may enhance therapeutic outcomes and survival rates in this patient population. Through this investigation, we aim to contribute valuable insights and offer a reference point for future research exploring the role of REC in cancer treatment.

## 2 Patients and methods

### 2.1 Case selection and baseline information

Participants for this study were identified from a cohort of patients with advanced NSCLC who were admitted to the affiliated hospital of Kunming University of Science and Technology between June 1, 2021, and December 30, 2022.

**Inclusion Criteria:**

1. **Diagnosis:** Patients who received their first pathological diagnosis of advanced NSCLC, categorized according to the 8th edition of the TNM classification system.
2. **Age Range:** Participants aged between 18 and 70 years were considered.
3. **Performance Status:** An Eastern Cooperative Oncology Group (ECOG) performance status score ranging from 0 to 2.
4. **Life Expectancy:** An anticipated survival duration of at least 6 months.
5. **Lesion Criteria:** Presence of at least one measurable lesion in the lung or mediastinum, as per the Response Evaluation Criteria In Solid Tumors (RECIST) version 1.1 standards.
6. **Consent:** Voluntary participation with signed informed consent.

**Exclusion Criteria:**

1. **Family Cancer History:** A familial history of cancer.
2. **Concurrent Malignancies:** Presence of other malignant tumors within the past 5 years.
3. **Autoimmune Diseases:** Active, known, or suspected autoimmune disease.
4. **Prior Immunotherapy:** Previous treatment with any T-cell co-stimulation or immune checkpoint inhibitors.
5. **Cardiovascular and Cerebrovascular Conditions:** Severe cardiovascular or cerebrovascular diseases.

These criteria were meticulously applied to ensure a well-defined study population and to control for confounding variables that could impact the evaluation of the therapeutic efficacy of rare earth carbonate in combination with chemoradiotherapy in late-stage NSCLC.

A total of 817 NSCLC patients were included, with the baseline data shown in **Table 1**. They were randomly divided into a control group (concurrent chemoradiotherapy, n=394) and an experimental group (concurrent chemoradiotherapy + REC, n=423), with both groups undergoing complete gene testing and collection of patient data and telephone follow-ups. Patients gave informed consent before treatment enrollment, approved by the Ethics Committee of Kunming University of Science and Technology, with approval number KMUST202102558456.”

**Table 1:** Patient baseline information.

Variable	*n*	Experimental group	Control group	X^2^ Value	P value
		*(n*=423)	(*n*=394)		
Sex(%)					
Male	712	372 (52.24)	340 (47.75)		1.000
Female	105	51 (48.57)	54 (51.42)		
Age(Year)					
≤60	752	382 (50.80)	370 (49.20)	5.913	0.419
>60	65	41 (63.08)	24 (36.92)		
Smoking history					
Yes	670	349 (52.09%)	321 (47.91%)	0.185	0.615
No	147	74 (42.52%)	73 (57.48%)		
Tumor location					
Left lobe	315	149 (47.30%)	166 (52.70%)	1.35	0.312
Right lobe	502	274 (54.58%)	228 (45.52%)		
T-staging					
T_1_	102	45 (44.11%)	57 (55.89%)	0.007	0.913
T_2_	215	109 (50.70%)	106 (49.30%)		
T_3_	367	192 (52.32%)	175 (47.68%)		
T_4_	133	77 (57.89%)	56 (42.11%)		
ECOG score (points)					
1	40	24 (60.00%)	16 (40.00%)	0.120	0.815
2	717	341 (47.56%)	376 (52.44%)		
3	60	29 (48.33%)	31 (51.67%)		

### 2.2 Treatment regimen

**Experimental group:**

**Radiation Therapy:** Utilizing a 10 MV-X medical linear accelerator for Intensity-Modulated Radiation Therapy (IMRT), delineating all tumor target areas and organs at risk. Conventional segmented radiation therapy was applied with a single dose of 6Gy, administered 3 times a week, for a total radiation dose of 30 Gy.

**Chemotherapy:** Etoposide injection 50 mg/m², administered continuously for 5 days, with a daily intravenous infusion lasting 4 hours; the therapy repeated every 28 days. Cisplatin injection 40 mg/m², administered once every 28 days.

**Combined with REC:** Daily intake of low-dose REC (0.05mg/kg).

**Control group:**

Adopted the same radiation and chemotherapy plan as the experimental group, but without combining with REC.

### 2.3 Standards for evaluating the effectiveness

Comparing the short-term clinical efficacy, adverse reactions, and six-month progression-free survival (PFS) of patients in both groups. Four weeks after concurrent treatment, comprehensive radiological and hematological examinations were completed, and the treatment effect on target lesions was assessed according to the RECIST 1.1 version. Adverse events were graded according to the Common Terminology Criteria for Adverse Events. The formulas for calculating the objective response rate (ORR) and disease control rate (DCR) are as follows:


ORR (%)=(Complete Remission+Partial Remission Total)×100%

DCR (%)=(Complete Remission+Partial Remission+Stable Total)×100%

### 2.4 Statistical methods

In the rigorous analysis of our collected data, we employed the statistical software SPSS 25.0. For the comparative analysis of qualitative data, we selected specific methodologies based on their appropriateness for our data types. The χ² test was used primarily for its efficacy in evaluating categorical data with larger sample sizes, while Fisher’s exact test was reserved for smaller sample sizes or instances where the χ² test’s assumptions were not met. To assess the PFS of patients, we employed the Kaplan-Meier estimator, renowned for its ability to generate survival probability estimates over time despite censored data. In identifying potential prognostic factors, Cox regression analysis was utilized due to its capacity to handle multiple covariates and its appropriateness for survival data, where the proportional hazards assumption applies. The decision to set the α level at 0.01, as opposed to the more common 0.05, was made to reduce the probability of type I errors (false positives), thereby ensuring a higher threshold for establishing statistical significance. This stringent criterion was deemed necessary due to the potential implications of our findings for clinical practice, where the accuracy and reliability of results are paramount. The adoption of this conservative α level implies that only those findings that demonstrate a very strong association (with a probability of less than 1% under the null hypothesis) are considered statistically significant, thereby bolstering the robustness and credibility of our conclusions.

## 3 Results

### 3.1 Baseline information

As delineated in **Table 1**, the foundational attributes of the participants enrolled in both the experimental and control cohorts are meticulously cataloged. Encompassing 394 individuals, the control assembly forms a critical comparison base to the slightly more populous experimental contingent, which is constituted of 423 patients. A rigorous statistical analysis yielded that the demographics and baseline characteristics distributed across both groups remained homogenized, failing to showcase any statistically significant differentiation (P>0.05). According to the TNM staging, the control group had 163 cases in stages T1-2 and 231 cases in stages T3-4; whereas, the experimental group had 154 cases in stages T1-2 and 269 cases in stages T3-4. There was no statistically significant difference in the TNM staging between the two groups (P>0.01).

### 3.2 Short-term efficacy

As shown in **Table 2**, a critical examination of the objective response rate (ORR) reveals a palpable dichotomy between the control and experimental factions, registering at 45.18% (178/394) and 89.83% (380/423) respectively — a disparity that unequivocally achieves statistical significance (p<0.01). Concurrently, the disease control rate (DCR) demonstrates a convergence between the groups, with figures standing at 85.28% (336/394) for the control and a marginally elevated 85.58% (362/423) for the experimental cohort; a comparison that, upon statistical scrutiny, fails to yield a significant divergence (P>0.01).

**Table 2:** The recent efficacy of the two groups of patients.

Type	*n*	CR	PR	SD	PD	ORR	DCR
Control group	394	0(0.00)	162 (41.12%)	175 (44.41%)	49 (12.44%)	178 (45.18%)	336 (85.28%)
Experimental group	423	0(0.00)	301 (71.16%)	94 (22.22%)	36 (8.51%)	380 (89.83%)	362 (85.58%)
X^2^ Value						4.626	0.868
P value						0.001	0.106

### 3.3 Adverse reactions

As shown in **Table 3**, the most common adverse reactions in the control group were lung infections (253/394, 64.21%), bone marrow suppression (196/394, 49.75%), and radiation pneumonia (102/394, 25.89%). Meanwhile, the experimental group most frequently experienced lung infections (172/423, 40.66%), bone marrow suppression (129/423, 30.49%), and radiation pneumonia (42/423, 31.91%). In the clinical trial under scrutiny, the experimental cohort exhibited an elevated propensity for incurring radiation-induced pneumonia relative to their counterparts in the control group, a distinction that did not, however, attain statistical significance at the established threshold (p>0.01). Remarkably, the experimental aggregation witnessed a diminution in the occurrence of both bone marrow suppression and pulmonary infections, a variation substantiated with statistical significance (p<0.01). It merits note that both investigative groups maintained a safety profile with no recorded incidents of adverse reactions exceeding a grade 3 severity, and conspicuously absent were any fatalities attributable to the treatment protocols.

**Table 3:** Comparison of adverse reactions between the two groups.

Type	*n*	Bone marrow suppression	Radiation pneumonia	Gastrointestinal Adverse Reactions	Abnormal liver function	Skin lesion	Heart failure	Electrolyte disturbance	Radiation esophagitis	Lung infections	Thyroiditis
Control group	394	196 (49.75%)	102 (25.89%)	41 (10.41%)	85 (21.57%)	24 (6.09%)	10 (2.54%)	26 (6.60%)	11 (2.79%)	253 (64.21%)	7 (1.78%)
Experimental group	423	129 (30.49%)	135 (31.91%)	28 (6.61%)	57 (13.47%)	51 (12.05%)	28 (6.62%)	14 (3.31%)	12 (2.84%)	172 (40.66%)	5 (1.18%)
X^2^ value		6.352	2.142		0.942					8.139	
P value		0.008	0.162	1.000	0.315	1.000	1.000	0.725	1.000	0.003	0.382

### 3.4 ECOG score

As shown in **Table 4**, the preliminary analysis of the patients’ ECOG performance statuses exhibited no discernible statistical distinction between the experimental and control cohorts prior to the therapeutic intervention (P>0.01). Subsequent to the treatment regimen, the experimental group manifested a marked enhancement in physical well-being, a fact underscored by the pronounced reduction in their ECOG scores relative to the control group — a disparity that was corroborated statistically (P<0.001). When engendering a juxtaposition of the pre-and post-treatment ECOG metrics, a trend of elevating scores became apparent in the control assembly, contrasting starkly with the diminishing scores evinced in the experimental contingent in the post-treatment phase.

**Table 4:** Comparison of ECOG scores between the two groups of patients before and after treatment.

Type	After treatment (points)			Before treatment (points)		
	1	2	3	1	2	3
*n*	386	181	249	40	717	60
Experimental group	327 (85.16%)	38 (20.99%)	28 (11.16%)	24 (60.0%)	341 (47.56%)	29 (48.33%)
Control group	59 (14.84%)	143 (79.01%)	221 (88.84%)	16 (40.0%)	376 (52.44%)	31 (51.67%)
*P* value	<0.001	<0.001	<0.001	0.355	0.415	0.812

### 3.5 Prognostic factors

Potential factors influencing prognosis were incorporated into the Cox hazard ratio model. The results demonstrated that whether or not REC was used in conjunction with the treatment was an independent risk factor affecting the six-month PFS of patients (HR=2.241, 95% CI: 2.192-2.336, P<0.01).

## 4 Discussion

Late-stage NSCLC accounts for approximately 28% to 33% of newly diagnosed NSCLC cases, of which about 35% have no opportunity for surgery at the time of initial diagnosis (27, 28). Concurrent chemoradiotherapy is the standard treatment plan. About 9% of patients progress during treatment, and 41% progress after treatment, resulting in a poor long-term prognosis (29–31). To augment the life expectancy of patients presently grappling with NSCLC, there has been a paradigm shift in contemporary clinical interventions toward a strategy that synergizes chemoradiotherapy with PD-1/PD-L1 inhibitors. PD-1 is predominantly localized on the membrane of T lymphocytes, a type of white blood cell integral to the immune response, while PD-L1 exhibits a primary expression on the surface of neoplastic cells and antigen-presenting cells (APC), serving a pivotal role in immunological pathways. The intricate molecular interaction whereby PD-L1 engages with PD-1 on tumor-infiltrating CD8+ T cells facilitates a significant dampening of the cytotoxic T lymphocyte proliferation and activation (32, 33). This phenomenon precipitates a formidable mechanism for tumor cells to exploit, culminating in a scenario of immune evasion, whereby the tumorous cells elude the vigilant surveillance of the immune system, thereby fostering an environment conducive to unchecked malignant progression (34, 35).

Radiation therapy can damage the DNA of tumor cells, causing the double-stranded DNA to unwind, thereby releasing tumor-associated antigens. These antigens can enhance the antigen presentation function of CD8+ T cells, further eliminating the remaining tumor cells. Moreover, radiation therapy can induce overexpression of PD-L1 dependent on CD8+ T cells, inversely regulating the tumor suppression effect downstream of CD8+ T cells. Immune checkpoint inhibitors can reactivate downstream responses. Chemotherapy can also regulate tumor T cell responses by enhancing tumor antigenicity, inducing immunogenic cell death of the tumor, downregulating immune suppressive microenvironment pathways, and enhancing effector T cell responses, thereby activating the tumor immune cycle (36–38). Based on the synergistic effects between chemoradiation and immunity, this study explored the possibility of concurrent chemoradiation therapy combined with REC in the treatment of late-stage NSCLC (39–41). This study showed that the ORR in the experimental group was better than that in the control group, indicating that the short-term treatment effect of combining REC was better (42–44). REC can reduce the expression of PD-1 and PD-L1 proteins, block their downstream immune suppressive signaling pathways, thereby preventing their impact on the immune function of the body, and subsequently preventing tumor immune escape. This study found that the 6-month PFS rate in the experimental group was better than that in the control group, and the results of the multifactor regression model suggested that whether REC is used in combination can independently predict the prognosis of patients (45–47). The above results are similar to those of Lin etal’s study (48), indicating that the combined use of chemoradiotherapy and REC can result in a better prognosis for patients.

This study compared the adverse reactions of two group plans, and no adverse events above level 3 were observed in either group. There were no treatment-related fatal events, and the post-treatment ECOG score in the experimental group improved compared to the pre-treatment score (49, 50). Common adverse reactions in the experimental group included lung infection, bone marrow suppression, and radiation pneumonitis. In the experimental cohort, the manifestation of radiation pneumonitis surpassed that observed in the control assembly, albeit without reaching a threshold of statistical significance to substantiate a meaningful divergence (50–52). Conversely, the comparative analysis revealed a notable decrement in the prevalence of both bone marrow suppression and pulmonary infections in the experimental contingent relative to the control group, a variation that attained statistical significance, thereby underscoring a potentially consequential differentiation in the adverse event profile between the two populations.This suggests that the overall safety of combined chemoradiotherapy and REC treatment is higher, with a lower risk of adverse reactions compared to chemoradiotherapy alone without REC treatment (53–55).The findings of this study, notably the improved ORR, PFS, and safety profile when incorporating sterilized REC with chemoradiotherapy, carry significant clinical implications for the treatment of late-stage NSCLC. Enhanced ORR indicates a higher proportion of patients experiencing a reduction in tumor size, suggesting that the combination therapy may offer a more effective initial treatment response. This could potentially translate into better symptom control and improved quality of life for patients. The extension in PFS observed in our study implies that patients could enjoy longer periods without disease progression, which is particularly crucial in the context of late-stage NSCLC where treatment options are limited. Importantly, the favorable safety profile of the combined treatment indicates a lower risk of severe adverse events, which is paramount in ensuring patient compliance and overall well-being during the course of treatment. Collectively, these findings suggest that integrating REC with chemoradiotherapy could represent a promising treatment modality for late-stage NSCLC patients, offering a balance between efficacy and safety. For clinicians, this could mean an expanded arsenal of therapeutic strategies, potentially leading to more individualized and effective treatment plans that prioritize both patient survival and quality of life.The mechanistic underpinnings of sterilized REC in cancer therapy, particularly in late-stage NSCLC, warrant a more comprehensive examination. At the molecular level, REC exhibits multifaceted interactions with cellular pathways that are pivotal in cancer progression and treatment response. Primarily, it has been postulated that REC induces oxidative stress within tumor cells, leading to apoptosis and cell cycle arrest. This is thought to occur through the generation of reactive oxygen species (ROS), which disrupt the redox balance within cancer cells, ultimately triggering cellular apoptosis pathways. Concurrently, REC demonstrates an affinity for binding to DNA, potentially leading to direct DNA damage and disruption of DNA repair mechanisms. This interference with the genomic integrity of cancer cells can sensitize them to the cytotoxic effects of both chemotherapy and radiation therapy. the immunomodulatory effects of REC are of particular interest. As highlighted in our findings, REC appears to attenuate the expression of immune checkpoint proteins such as PD-1 and PD-L1. This inhibition plays a crucial role in reversing immune evasion strategies employed by tumor cells. By blocking these pathways, REC potentially reactivates cytotoxic T lymphocytes, enhancing the immune system’s ability to target and destroy cancer cells. This mechanism dovetails with the effects of chemoradiotherapy, which, by inducing immunogenic cell death, can expose tumor antigens and further potentiate an immune response. Additionally, REC might influence the tumor microenvironment, possibly by modulating the secretion of cytokines and chemokines that govern the recruitment and activity of immune cells. The therapeutic efficacy of REC in late-stage NSCLC seems to stem from a synergistic interplay between its direct cytotoxic effects, DNA binding properties, and immunomodulatory actions. These multifactorial mechanisms position REC as a promising adjunct to conventional chemoradiotherapy, potentially enhancing treatment outcomes through a comprehensive attack on cancer cells and their supportive microenvironment.

Treatment-related pneumonitis is a common adverse reaction in lung cancer. Chemoradiotherapy can increase capillary permeability, induce the deposition of pro-inflammatory cytokines and chemokines, and release reactive oxygen species, leading to alveolitis. Meanwhile, REC inhibiting PD-1 and PD-L1 can exacerbate this response. In this study, the incidence of radiation pneumonitis in the experimental and control groups were 31.91% and 25.89%, respectively, with the experimental group showing a 23.25% increase compared to the control group (38, 56–58). A study by Tong etal (59) found that the combination of chemoradiotherapy and PD-1/PD-L1 inhibitors increased the incidence of pneumonitis by 25.19% compared to chemoradiotherapy alone, which is consistent with this study. The differences in the incidence rates of pneumonitis across various studies might be related to the specific staging of the patients included, different physical conditions, and the use of different anti-PD-1 or anti-PD-L1 drugs (60–62). Lustberg etal’s study (63) exploring predictive parameters for pneumonitis found that a decline in alveolar diffusion function post-treatment is a risk factor for the occurrence of treatment-related pneumonitis, and it is related to the response to subsequent glucocorticoid treatment, which can be used to predict the onset and treatment outcome of pneumonitis. Therefore, monitoring the changes in lung function before and after treatment with REC can help identify the onset of pneumonitis early and guide further treatment.

Several studies have found that patients using REC often experience treatment-related pneumonitis after the first dose. The potential mechanism could be the inhibition of PD-1/PD-L1 leading to an increase in immune cell activity, thereby increasing the expression of type II helper T cell (Th2) cytokine interleukin-4 (IL-4), eventually causing immune pneumonitis (64–66). To mitigate this risk, clinical practice commonly involves combining REC with monoclonal antibodies against interleukin. While combining chemoradiotherapy with REC shows promise in treating late-stage NSCLC, it necessitates careful management of radiation pneumonitis. Future strategies should include preventive measures like early use of anti-inflammatory agents, personalized treatment plans based on patient risk factors, regular monitoring for early signs of pneumonitis, and exploration of combination therapies targeting pneumonitis pathogenesis. This balanced approach aims to optimize therapeutic benefits while minimizing adverse effects, ultimately enhancing patient outcomes.

The primary strength of this study resides in the execution of a comparative trial examining the therapeutic effects of concurrent chemoradiation therapy combined with sterilized REC in patients with advanced NSCLC. This was achieved through a prospective cohort study, incorporating a diverse sample range. Additionally, the study explored the underlying mechanisms of cancer treatment involving this combination, setting a foundation for future investigations into REC-based adjuvant therapies in cancer treatment. However, this research is not without its limitations. A notable limitation is that all participants included in this study were of Asian descent, potentially introducing bias due to the homogeneity of the population sample. Recognizing this limitation, we are committed to expanding our research scope. In forthcoming studies, we plan to conduct international multicenter research to assess the therapeutic effects of concurrent chemoradiation therapy combined with REC. These studies are intended to encompass a broader demographic, including populations from North America, Europe, and other Asian countries, thereby mitigating the limitations encountered in the present study. We aim to initiate this global collaboration within the next two years, ensuring diverse participation across continents. Furthermore, steps will be taken to control for potential bias, including stratified random sampling and implementing standardized protocols across all participating centers. This approach is anticipated to yield more generalizable results, thereby enriching our understanding of REC’s role in treating advanced NSCLC globally.

## 5 Conclusion

The addition of sterilized REC to concurrent chemoradiotherapy in late-stage NSCLC patients significantly enhances short-term therapeutic outcomes and prognosis compared to chemoradiotherapy alone. The study demonstrated a notable improvement in objective response rates and a reduction in adverse reactions, particularly in bone marrow suppression and pulmonary infections, in the experimental group receiving REC. Additionally, patients treated with REC exhibited better physical well-being post-treatment, as evidenced by lower ECOG scores. These findings highlight REC’s potential as a valuable adjunct in the treatment of advanced NSCLC, offering improved efficacy and safety.

## Data availability statement

The original contributions presented in the study are included in the article/supplementary material. Further inquiries can be directed to the corresponding authors.

## Ethics statement

The patient in our research has signed the informed consent. This study was designed in accordance with the Declaration of Helsinki and approved by the ethics committee of Kunming University of Science and Technology. Approval number: KMUST202102558456. The studies were conducted in accordance with the local legislation and institutional requirements. The participants provided their written informed consent to participate in this study.

## Author contributions

QC: Conceptualization, Data curation, Formal Analysis, Funding acquisition, Investigation, Methodology, Project administration, Resources, Software, Supervision, Validation, Visualization, Writing – original draft, Writing – review & editing. XY: Conceptualization, Resources, Data curation, Software, Formal analysis, Supervision, Funding Acquisition, Validation, Investigation, Visualization, Methodology, Writing – Original Draft, Project administration, Writing – Review & Editing. XW: Conceptualization, Data curation, Formal Analysis, Funding acquisition, Investigation, Methodology, Project administration, Resources, Software, Supervision, Validation, Visualization, Writing – original draft, Writing – review & editing. QZ: Conceptualization, Data curation, Formal Analysis, Funding acquisition, Investigation, Methodology, Project administration, Resources, Software, Supervision, Validation, Visualization, Writing – original draft, Writing – review & editing. JG: Conceptualization, Data curation, Formal Analysis, Funding acquisition, Investigation, Methodology, Project administration, Resources, Software, Supervision, Validation, Visualization, Writing – original draft, Writing – review & editing. YC: Conceptualization, Data curation, Formal Analysis, Funding acquisition, Investigation, Methodology, Project administration, Resources, Software, Supervision, Validation, Visualization, Writing – original draft, Writing – review & editing. YY: Conceptualization, Data curation, Formal Analysis, Funding acquisition, Investigation, Methodology, Project administration, Resources, Software, Supervision, Validation, Visualization, Writing – original draft, Writing – review & editing. JS: Conceptualization, Data curation, Formal Analysis, Funding acquisition, Investigation, Methodology, Project administration, Resources, Software, Supervision, Validation, Visualization, Writing – original draft, Writing – review & editing. YQ: Conceptualization, Data curation, Formal Analysis, Funding acquisition, Investigation, Methodology, Project administration, Resources, Software, Supervision, Validation, Visualization, Writing – original draft, Writing – review & editing. GC: Conceptualization, Data curation, Formal Analysis, Funding acquisition, Investigation, Methodology, Project administration, Resources, Software, Supervision, Validation, Visualization, Writing – original draft, Writing – review & editing.

## References

[1] Schmidt DR Patel R Kirsch DG Lewis CA Vander Heiden MG Locasale JW. Metabolomics in cancer research and emerging applications in clinical oncology. CA: Cancer J Clin (2021) 71(4):333–58. doi: 10.3322/caac.21670 PMC8298088 33982817

[2] Gkountakos A Delfino P Lawlor RT Scarpa A Corbo V Bria E. Harnessing the epigenome to boost immunotherapy response in non-small cell lung cancer patients. Ther Adv Med Oncol (2021) 13:17588359211006947. doi: 10.1177/17588359211006947 34104224 PMC8161860

[3] Ilnytskyy Y Petersen L McIntyre JB Konno M D'Silva A Dean M. Genome-wide detection of chimeric transcripts in early-stage non-small cell lung cancer. Cancer Genomics Proteomics (2023) 20(5):417–32. doi: 10.21873/cgp.20394 PMC10464939 37643782

[4] Smolle E Leithner K Olschewski H. Oncogene addiction and tumor mutational burden in non-small-cell lung cancer: clinical significance and limitations. Thorac Cancer (2020) 11(2):205–15. doi: 10.1111/1759-7714.13246 PMC6997016 31799812

[5] Abu Al Karsaneh O Al Anber A ALQudah M Al-Mustafa ALQudah M AlMa'aitah S H. Prevalence and clinicopathological associations of HER2 expression in non-small cell lung cancer: a retrospective study in Jordanian patients. Diagn Pathol (2023) 18(1):1–13. doi: 10.1186/s13000-023-01364-2 37340403 PMC10283286

[6] Suster DI Mino-Kenudson M. Molecular pathology of primary non-small cell lung cancer. Arch Med Res (2020) 51(8):784–98. doi: 10.1016/j.arcmed.2020.08.004 32873398

[7] John T Taylor A Wang H Eichinger C Freeman C Ahn MJ. Uncommon EGFR mutations in non-small-cell lung cancer: A systematic literature review of prevalence and clinical outcomes. Cancer Epidemiol (2022) 76:102080. doi: 10.1016/j.canep.2021.102080 34922050

[8] Shah HJ Ruppell E Bokhari R Aland P Lele VR Ge C. Current and upcoming radionuclide therapies in the direction of precision oncology: A narrative review. Eur J Radiol Open (2023) 10:100477. doi: 10.1016/j.ejro.2023.100477 36785643 PMC9918751

[9] Wang Y Ding X Liu B Li M Chang Y Shen H. ETV4 overexpression promotes progression of non–small cell lung cancer by upregulating PXN and MMP1 transcriptionally. Mol Carcinogenesis (2020) 59(1):73–86. doi: 10.1002/mc.23130 31670855

[10] Huang G Li W Meng M Ni Y Han X Wang J. Synchronous microwave ablation combined with cisplatin intratumoral chemotherapy for large non-small cell lung cancer. Front Oncol (2022) 12:955545. doi: 10.3389/fonc.2022.955545 35965525 PMC9369018

[11] Jovanoski N Abogunrin S Di Maio D Belleli R Hudson P Bhadti S. Systematic literature review to identify cost and resource use data in patients with early-stage non-small cell lung cancer (NSCLC). PharmacoEconomics (2023), 1–16. doi: 10.1007/s40273-023-01295-2 PMC10570243 37389802

[12] Pankova OV Rodionov EO Miller SV Tuzikov SA Tashireva LA Gerashchenko TS. Neoadjuvant chemotherapy combined with intraoperative radiotherapy is effective to prevent recurrence in high-risk non-small cell lung cancer (NSCLC) patients. Trans Lung Cancer Res (2020) 9(4):988. doi: 10.21037/tlcr-19-719 PMC7481627 32953479

[13] Nardone V Belfiore MP De Chiara M De Marco G Patanè V Balestrucci G. CARdioimaging in lung cancer patiEnts undergoing radical radioTherapy: CARE-RT trial. Diagnostics (2023) 13(10):1717. doi: 10.3390/diagnostics13101717 37238201 PMC10217672

[14] Zayed S Louie AV Breadner DA Palma DA Correa RJM. Radiation and immune checkpoint inhibitors in the treatment of oligometastatic non-small-cell lung cancer: a practical review of rationale, recent data, and research questions. Ther Adv Med Oncol (2023) 15:17588359231183668. doi: 10.1177/17588359231183668 37435562 PMC10331344

[15] Parsi M Vivacqua RJ. Synchronous multiple primary cancers of the lung: the rare association of non-small cell carcinoma with a carcinoid tumor. Cureus (2020) 12(8). doi: 10.7759/cureus.9888 PMC7502415 32968554

[16] Hong E Liu L Bai L Xia C Gao L Zhang L. Control synthesis, subtle surface modification of rare-earth-doped upconversion nanoparticles and their applications in cancer diagnosis and treatment. Mater Sci Eng: C (2019) 105:110097. doi: 10.1016/j.msec.2019.110097 31546381

[17] Ruiyi L Zaijun L Xiulan S Jan J Lin L Zhiguo G. Graphene quantum dot-rare earth upconversion nanocages with extremely high efficiency of upconversion luminescence, stability and drug loading towards controlled delivery and cancer theranostics. Chem Eng J (2020) 382:122992. doi: 10.1016/j.cej.2019.122992

[18] Chen Z Liu G Zhang X Sui J Dong X Yu W. Synthesis of multifunctional rare-earth fluoride/Ag nanowire nanocomposite for efficient therapy of cancer. Mater Sci Eng: C (2019) 104:109940. doi: 10.1016/j.msec.2019.109940 31500050

[19] Hossain MK Khan MI El-Denglawey A. A review on biomedical applications, prospects, and challenges of rare earth oxides. Appl Mater Today (2021) 24:101104. doi: 10.1016/j.apmt.2021.101104

[20] Bulin AL Broekgaarden M Chaput F Baisamy V Garrevoet J Busser B. Radiation dose-enhancement is a potent radiotherapeutic effect of rare-earth composite nanoscintillators in preclinical models of glioblastoma. Adv Sci (2020) 7(20):2001675. doi: 10.1002/advs.202001675 PMC7578894 33101867

[21] Xia Y Zhang X Sun D Gao Y Zhang X Wang L. Effects of water-soluble components of atmospheric particulates from rare earth mining areas in China on lung cancer cell cycle. Particle Fibre Toxicol (2021) 18(1):1–23. doi: 10.1186/s12989-021-00416-z PMC8330054 34340691

[22] Ding B Sheng J Zheng P Li C Li D Cheng Z. Biodegradable upconversion nanoparticles induce pyroptosis for cancer immunotherapy. Nano Lett (2021) 21(19):8281–9. doi: 10.1021/acs.nanolett.1c02790 34591494

[23] Dot T Le M Vo N Nguyen T Tran T Phung T. Cancer stem cell target labeling and efficient growth inhibition of CD133 and PD-L1 monoclonal antibodies double conjugated with luminescent rare-earth Tb3+ nanorods. Appl Sci (2020) 10(5):1710.

[24] Fan Q Cui X Guo H Xu Y Zhang G Peng B. Application of rare earth-doped nanoparticles in biological imaging and tumor treatment. J Biomater Appl (2020) 35(2):237–63. doi: 10.1177/0885328220924540 32423319

[25] Cai Y Chen X Si J Mou X Dong X. All-in-one nanomedicine: multifunctional single-component nanoparticles for cancer theranostics. Small (2021) 17(52):2103072. doi: 10.1002/smll.202103072 34561968

[26] Wu M Xue Y Li N Zhao H Lei B Wang M. Tumor-microenvironment-induced degradation of ultrathin gadolinium oxide nanoscrolls for magnetic-resonance-imaging-monitored, activatable cancer chemotherapy. Angewandte Chemie Int Edit (2019) 58(21):6880–5. doi: 10.1002/anie.201812972 30680857

[27] Miller KD Nogueira L Mariotto AB Rowland JH Yabroff KR Alfano CM. Cancer treatment and survivorship statistics, 2019. CA: Cancer J Clin (2019) 69(5):363–85. doi: 10.3322/caac.21565 31184787

[28] Ellison LF Saint-Jacques N. Five-year cancer survival by stage at diagnosis in Canada. Health Rep (2023) 34(1):3–15. 10.25318/82-003-x202300100001-eng 36716075

[29] Song Q Li J Xiong Q Long YP Yang B Dong ZH. Neoadjuvant immunotherapy plus chemotherapy and adjuvant targeted therapy in ALK-positive non-small-cell lung cancer. Immunotherapy (2023) 0). doi: 10.2217/imt-2022-0302 37254687

[30] Poettgen C Gkika E Stahl M Abu Jawad J Gauler T Kasper S. Dose-escalated radiotherapy with PET/CT based treatment planning in combination with induction and concurrent chemotherapy in locally advanced (uT3/T4) squamous cell cancer of the esophagus: mature results of a phase I/II trial. Radiat Oncol (2021) 16(1):1–11. doi: 10.1186/s13014-021-01788-4 33757534 PMC7988964

[31] Zheng L Zhou ZR Shi M Chen H Yu QQ Yang Y. Nomograms for predicting progression-free survival and overall survival after surgery and concurrent chemoradiotherapy for glioblastoma: a retrospective cohort study. Ann Trans Med (2021) 9(7). doi: 10.21037/atm-21-673 PMC8105825 33987269

[32] Garrido F Aptsiauri N. Cancer immune escape: MHC expression in primary tumours versus metastases. Immunology (2019) 158(4):255–66. doi: 10.1111/imm.13114 PMC6856929 31509607

[33] Rosenthal R Cadieux EL Salgado R Bakir MA Moore DA Hiley CT. Neoantigen-directed immune escape in lung cancer evolution. Nature (2019) 567(7749):479–85. doi: 10.1038/s41586-019-1032-7 PMC6954100 30894752

[34] Chen M Wang P Jiang D Bao Z Quan H. Platelet membranes coated gold nanocages for tumor targeted drug delivery and amplificated low-dose radiotherapy. Front Oncol (2021) 11:793006. doi: 10.3389/fonc.2021.793006 34900745 PMC8651991

[35] Nickoloff JA Taylor L Sharma N Kato TA. Exploiting DNA repair pathways for tumor sensitization, mitigation of resistance, and normal tissue protection in radiotherapy. Cancer Drug Resist (2021) 4(2):244. doi: 10.20517/cdr.2020.89 34337349 PMC8323830

[36] Pan W Gong S Wang J Yu L Chen Y Li N. A nuclear-targeted titanium dioxide radiosensitizer for cell cycle regulation and enhanced radiotherapy. Chem Commun (2019) 55(56):8182–5. doi: 10.1039/C9CC01651A 31241064

[37] Yu X Ma H Xu G Liu Z. Radiotherapy assisted with biomaterials to trigger antitumor immunity. Chin Chem Lett (2022) 33(9):4169–74. doi: 10.1016/j.cclet.2022.02.049

[38] Cao Q Wu X Chen Y Wei Q You Y Qiang Y. The impact of concurrent bacterial lung infection on immunotherapy in patients with non-small cell lung cancer: a retrospective cohort study. Front Cell Infect Microbiol (2023) 13. doi: 10.3389/fcimb.2023.1257638 PMC10497767 37712056

[39] Patyal M Kaur K Bala N Gupta N Malik AK. Innovative Lanthanide Complexes: Shaping the future of cancer/tumor Chemotherapy. J Trace Elem Med Biol (2023), 127277. doi: 10.1016/j.jtemb.2023.127277 37572546

[40] Cao Q Zhang Q Zhou KX Li YX Yu Y He ZX. Lung cancer screening study from a smoking population in Kunming. Eur Rev Med Pharmacol Sci (2022) 26(19). 10.26355/eurrev_202210_29894 36263557

[41] Peng X Gao S Zhang JL. Gallium (III) complexes in cancer chemotherapy. Eur J Inorgan Chem (2022) 2022(6):e202100953. doi: 10.1002/ejic.202100953

[42] Zhang H Lv Z Xue D Zhang T Jin L Cao Y. A tumor microenvironment-responsive theranostic agent for synergetic therapy of disulfiram-based chemotherapy and chemodynamic therapy. J Phys Chem Lett (2021) 12(44):10880–5. doi: 10.1021/acs.jpclett.1c03184 34730355

[43] Qiang C Qi Z Yi Q. Mechanisms of p2x7 receptor involvement in pain regulation: a literature review. Acta Med Mediterr (2022) 38(2):1187–94.

[44] Chen W Xie Y Wang M Li C. Recent advances on rare earth upconversion nanomaterials for combined tumor near-infrared photoimmunotherapy. Front Chem (2020) 8:596658. doi: 10.3389/fchem.2020.596658 33240857 PMC7677576

[45] Xu Y Song G Xie S Jiang W Chen X Chu M. The roles of PD-1/PD-L1 in the prognosis and immunotherapy of prostate cancer. Mol Ther (2021) 29(6):1958–69. doi: 10.1016/j.ymthe.2021.04.029 PMC8178461 33932597

[46] Somasundaram R Connelly T Choi R Choi H Samarkina A Li L. Tumor-infiltrating mast cells are associated with resistance to anti-PD-1 therapy. Nat Commun (2021) 12(1):346. doi: 10.1038/s41467-020-20600-7 33436641 PMC7804257

[47] Kataria S Qi J Lin CW Li Z Dane EL Iyer AM. Noninvasive *in vivo* imaging of T-cells during cancer immunotherapy using rare-earth nanoparticles. ACS nano (2023). doi: 10.1021/acsnano.3c03882 37676036

[48] Lin B Wang Y Zhao K Lü WD Hui X Ma Y. Exosome-based rare earth nanoparticles for targeted *in situ* and metastatic tumor imaging with chemo-assisted immunotherapy. Biomater Sci (2022) 10(3):744–52. doi: 10.1039/D1BM01809D 34940770

[49] Shaverdian N Yeboa DN Gardner L Harari PM Liao K McCloskey S. Nationwide survey of patients’ perspectives regarding their radiation and multidisciplinary cancer treatment experiences. J Oncol Pract (2019) 15(12):e1010–7. doi: 10.1200/JOP.19.00376 31747336

[50] Dilalla V Chaput G Williams T Sultanem K. Radiotherapy side effects: integrating a survivorship clinical lens to better serve patients. Curr Oncol (2020) 27(2):107–12. doi: 10.3747/co.27.6233 PMC7253739 32489253

[51] Wu X Zhou Z Cao Q Chen Y Gong J Zhang Q. Reprogramming of treg cells in the inflammatory microenvironment during immunotherapy: A literature review. Front Immunol (2023) 14:1268188. doi: 10.3389/fimmu.2023.1268188 37753092 PMC10518452

[52] Cao Q Zhang Q Chen YQ Fan AD Zhang XL. Risk factors for the development of hepatocellular carcinoma in Chengdu: a prospective cohort study. Eur Rev Med Pharmacol Sci (2022) 26(24). 10.26355/eurrev_202212_30696 36591853

[53] Yang L Xu X Liu Q. Establishment of a risk prediction model for pulmonary infection in patients with advanced cancer. Appl Bionics Biomech (2022) 2022. doi: 10.1155/2022/6149884 PMC9170436 35677196

[54] Lyu J Shi A Li T Li J Zhao R Zhu S. Effects of enteral nutrition on patients with oesophageal carcinoma treated with concurrent chemoradiotherapy: a prospective, multicentre, randomised, controlled study. Front Oncol (2022) 12:839516. doi: 10.3389/fonc.2022.839516 35280748 PMC8914079

[55] Wang C Zhu S Miao C Wang Y Chen J Yuan S. Safety and efficacy of pegylated recombinant human granulocyte colony-stimulating factor during concurrent chemoradiotherapy for small-cell lung cancer: a retrospective, cohort-controlled trial. BMC Cancer (2022) 22(1):542. doi: 10.1186/s12885-022-09644-8 35562713 PMC9107159

[56] Hu Q Wang S Ma L Sun Z Liu Z Deng S. Radiological assessment of immunotherapy effects and immune checkpoint-related pneumonitis for lung cancer. J Cell Mol Med (2023). doi: 10.1111/jcmm.17895 PMC10902575 37525480

[57] You Y Chen Y Zhang Q Yan N Ning Y Cao Q. Muscle quality index is associated with trouble sleeping: a cross-sectional population based study. BMC Public Health (2023) 23(1):489. doi: 10.1186/s12889-023-15411-6 36918831 PMC10012435

[58] Barrón F Sánchez R Arroyo-Hernández M Blanco C Zatarain-Barrón ZL Catalán R. Risk of developing checkpoint immune pneumonitis and its effect on overall survival in non-small cell lung cancer patients previously treated with radiotherapy. Front Oncol (2020) 10:570233. doi: 10.3389/fonc.2020.570233 33117699 PMC7550759

[59] Tong Q Wu Y Liu D Dong M. Incidence risk of PD-1/PD-L1–related pneumonia and diarrhea in non-small cell lung cancer (NSCLC) patients: a systematic review and meta-analysis of randomized controlled trials. Eur J Clin Pharmacol (2021) 77:1079–88. doi: 10.1007/s00228-020-03083-9 33564898

[60] Chen X Nie J Dai L Hu W Zhang J Han J. Immune-related adverse events and their association with the effectiveness of PD-1/PD-L1 inhibitors in non-small cell lung cancer: a real-world study from China. Front Oncol (2021) 11:607531. doi: 10.3389/fonc.2021.607531 33747922 PMC7973369

[61] Cao Q Wang Q Wu X Zhang Q Huang J Chen Y. A literature review: mechanisms of antitumor pharmacological action of leonurine alkaloid. Front Pharmacol (2023) 14:1272546. doi: 10.3389/fphar.2023.1272546 37818195 PMC10560730

[62] Su Q Zhu EC Wu J Li T Hou YL Wang DY. Risk of pneumonitis and pneumonia associated with immune checkpoint inhibitors for solid tumors: a systematic review and meta-analysis. Front Immunol (2019) 10:108. doi: 10.3389/fimmu.2019.00108 30778352 PMC6369169

[63] Lustberg MB Kuderer NM Desai A Bergerot C Lyman GH. Mitigating long-term and delayed adverse events associated with cancer treatment: implications for survivorship. Nat Rev Clin Oncol (2023), 1–16. doi: 10.1038/s41571-023-00776-9 PMC10211308 37231127

[64] Sun Q Hong Z Zhang C Wang L Han Z Ma D. Immune checkpoint therapy for solid tumours: clinical dilemmas and future trends. Signal Transduct Targeted Ther (2023) 8(1):320. doi: 10.1038/s41392-023-01522-4 PMC10460796 37635168

[65] Shi R Tang YQ Miao H. Metabolism in tumor microenvironment: Implications for cancer immunotherapy. MedComm (2020) 1(1):47–68. doi: 10.1002/mco2.6 34766109 PMC8489668

[66] Wang J Long R Han Y. The role of exosomes in the tumour microenvironment on macrophage polarisation. Biochim Biophys Acta (BBA)-Reviews Cancer (2022), 188811. doi: 10.1016/j.bbcan.2022.188811 36208648

